# Genetic Variation in the Chemical Components of *Eucalyptus globulus* Wood

**DOI:** 10.1534/g3.111.000372

**Published:** 2011-07-01

**Authors:** Desmond J. Stackpole, René E. Vaillancourt, Ana Alves, José Rodrigues, Brad M. Potts

**Affiliations:** * School of Plant Sciences and Cooperative Research Centre for Forestry, University of Tasmania, Hobart 7001, Tasmania, Australia; † Scion, Rotorua 3046, New Zealand; ‡ Forestry and Forest Products Center, Tropical Research Institute of Portugal (IICT), Tapada Ajuda, 1349-017 Lisboa, Portugal

**Keywords:** tree improvement, wood chemicals, adaptation, lignin, cellulose, extractives, syringyl, guaiacyl

## Abstract

Despite the ecological and economic importance of lignin and other wood chemical components, there are few studies of the natural genetic variation that exists within plant species and its adaptive significance. We used models developed from near infra-red spectroscopy to study natural genetic variation in lignin content and monomer composition (syringyl-to-guaiacyl ratio [S/G]) as well as cellulose and extractives content, using a 16-year-old field trial of an Australian tree species, *Eucalyptus globulus*. We sampled 2163 progenies of 467 native trees from throughout the native geographic range of the species. The narrow-sense heritability of wood chemical traits (0.25–0.44) was higher than that of growth (0.15), but less than wood density (0.51). All wood chemical traits exhibited significant broad-scale genetic differentiation (*Q_ST_* = 0.34–0.43) across the species range. This differentiation exceeded that detected with putatively neutral microsatellite markers (*F_ST_* = 0.09), arguing that diversifying selection has shaped population differentiation in wood chemistry. There were significant genetic correlations among these wood chemical traits at the population and additive genetic levels. However, population differentiation in the S/G ratio of lignin in particular was positively correlated with latitude (*R^2^* = 76%), which may be driven by either adaptation to climate or associated biotic factors.

Forests occupy 30% of the world’s terrestrial surface (FAO 2007) and are key terrestrial carbon stores, much of which is from wood (Sedjo 1993). Wood derived from natural and planted forests is also the basis of renewable energy and industrial production systems for products such as timber and pulp, worth more than $327 billion US in annual trade (FAO 2007). Wood is the fibrous material in the trunk of trees under the bark, which is composed of a complex mix of plant polymers. The most important quantitatively is cellulose, followed by lignin, hemicelluloses, and then extractives (Walker 2006). Cellulose fibers are deposited on cell walls along with lignin during the process of wood formation as cells expand following differentiation at the cambium (Walker 2006). Cellulose gives strength to the cell walls (Turner and Somerville 1997). Lignin supports the cellulose fibers, provides the hydrophobic surfaces in vessels essential for water conduction (Plomion *et al.* 2001), and also has roles in defense against wood eaters and pathogens (Coleman *et al.* 2008; Salmore and Hunter 2001).

The evolution of lignin biosynthesis has been fundamental to the adaptation to the terrestrial environment (Weng and Chapple 2010), and the proportion of lignin in wood varies markedly between species (15%–36%) (Zobel and Van Buijtenen 1989). Lignin is constructed of three monolignol monomers, hydroxyphenyl (H), guaiacyl (G), and syringyl (S), with the proportion and location of the different monomers varying between and within species (Anterola and Lewis 2002). These monomers are synthesized in the cytoplasm, but lignin is formed when they are polymerized at the site of deposition (Lewis and Yamamoto 1990). The presence of methoxyl groups attached to the benzene ring of the lignin monomer increases the reactivity of the lignin to natural or artificial delignification agents (Pinto *et al.* 2002). As S has two methoxyl groups attached to the benzene ring, it has higher reactivity than G, which has only one methoxyl group. H is the least reactive having no methoxyl groups. Gymnosperm lignin is almost entirely composed of G with only a minor proportion of H and S (Alves *et al.* 2006; Campbell and Sederoff 1996; Godoy *et al.* 2007; Walker 2006), while woody angiosperms have H in trace amounts (Rencoret *et al.* 2008). In woody angiosperms, the ratio of S to G monomers (S/G) varies between species, provenance, and also between cell type within a tree (Pinto *et al.* 2002; Rodrigues *et al.* 1999; Rodrigues *et al.* 2001). Wood also contains extractives, a diverse group of nonstructural compounds that are mainly involved in chemical and physical defenses of living and dead wood (Boddy 2001). Extractives are present in sapwood, but preferentially deposited in the heartwood (Taylor *et al.* 2002). Resin acids predominate in the extractives of conifer heartwood, whereas a wide range of compounds occur in angiosperms, although in any one species the range is reduced (Gutierrez *et al.* 1999).

Planted forests comprise an increasing proportion of the world’s forests and now provide nearly half of the global wood production (FAO 2007). Short-rotation tree crops such as eucalypt plantations, are the feed stocks not only of the pulp and paper industry (Clarke 2009; Cotterill and Brolin 1997; Paues 1999), but are also seen as the basis of new industries replacing the use of fossil hydrocarbons for energy and industrial organic chemicals (Bozell 2010). In the case of pulp production, the strongest and highest quality pulp is produced by chemical delignification using powerful bases (the kraft process) leaving the cellulose fibers relatively undamaged for reconstitution as paper (Clarke 2009). Angiosperm wood with high S/G tends to be easier to delignify as per unit weight of dry wood it consumes less chemical and energy and produces higher pulp yield (Rencoret *et al.* 2007). Extractives may interfere with the efficiency of delignifying chemicals (Wallis *et al.* 1996) and also have adverse effects on the pulping process as they accumulate in industrial conduits which are costly to clean (Hillis and Sumimoto 1989).

Breeding objectives for biomass crops intended for delignification could conceivably include reduced total lignin content or increased S/G (Bose *et al.* 2009). Conversely, other processes or products might require higher lignin content or lower S/G (Clarke 2009). Similarly, adaptation of the tree to abiotic or biotic environmental stresses may be impacted by changes in lignin content or composition, and processing objectives may or may not be aligned with requirements for plantations to be adapted to current or future environments. The extent to which breeders can directly or indirectly change wood chemical composition through selection will depend upon its quantitative genetic architecture, including the levels of additive genetic variation in the population, its heritability, and correlations between chemical components and other traits (Falconer and Mackay 1996). However, despite the economic and ecological importance of lignin and other wood chemicals, as well as decades of biotechnological research on specific genotypes (Coleman *et al.* 2008; Weng and Chapple 2010), the basic quantitative genetic architecture of these traits across the gene pool of any woody angiosperm species is poorly known. This has been mainly due to the high cost of measuring lignin, which has prevented the acquisition of the large sample sizes required to obtain robust and accurate genetic parameter estimates (Falconer and Mackay 1996). Quantification of wood chemical constituents is notoriously difficult, and the different methods used all have limitations (Anterola and Lewis 2002; Hatfield and Fukushima 2005). However, rapid and inexpensive near-infrared (NIR) reflectance chemometric methods have been developed that allow the prediction of chemical composition from NIR spectra (Schimleck *et al.* 2000; Workman 1992). NIR has been applied to large-scale studies of conifer species to estimate genetic parameters for lignin content and quality (Da Silva Perez *et al.* 2007), but has not been applied in large-scale studies of the genetic architecture of woody angiosperms.

In this study, a large base population trial of open-pollinated *Eucalyptus globulus* was used to study the quantitative genetic architecture of wood chemical components (lignin, S/G, extractives, and cellulose) and their genetic correlation with traits under artificial selection (growth, wood density, and pulp yield; Stackpole *et al.* 2010b). Having studied the geographic variation in the wood chemicals across the geographic range of the species, we provide evidence that there is a signature of natural selection acting on these traits and discuss the potential drivers of genetic divergence.

## Materials and Methods

### Study system

**Figure 1:**
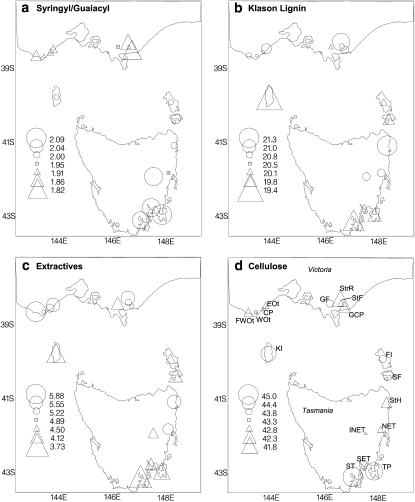
Geographic distribution of subrace means for syringyl/guaiacyl ratio (S/G), Klason lignin, extractives and cellulose content in *Eucalyptus globulus* grown in a common environment field trial. The larger the circle or triangle, the more the subrace mean deviates above or below the mid range value. Subrace codes are as follows: TP - Tasman Peninsula; SET - South-eastern Tasmania; INET - Inland North-eastern Tasmania; ST - Southern Tasmania; SF - Southern Furneaux; StH - St Helens; FI - Flinders Island; KI - King Island; GF - Gippsland Foothills; NET - North-eastern Tasmania; StF - Strzelecki Foothills; WOt -Western Otways; CP - Cape Patton; EOt - Eastern Otways; FWOt - Far West Otways; StrR - Strzelecki Ranges; GCP - Gippsland Coastal Plain.

*Eucalyptus globulus* Labill. (tasmanian blue gum *sensu* Brooker (2000), otherwise known as *E. globulus* ssp. *globulus*, Kirkpatrick,) is the main hardwood species grown in temperate Mediterranean climates across the globe (Potts *et al.* 2004). In its native range in south eastern Australia (Figure 1), it is often a dominant of coastal forests that typically grows 15 to 60 m tall (Williams and Potts 1996). The species is genetically diverse with geographic races showing broad-scale differences in numerous quantitative traits (Dutkowski and Potts 1999), many of which are presumably adaptive (*e.g.*, frost tolerance; Tibbits *et al.* 2006; drought tolerance, Dutkowski 1995; Toro *et al.* 1998). Microsatellite analysis shows that contiguous races are more similar to one another than distant ones (Steane *et al.* 2006). *E. globulus* has a mixed mating system, and its open-pollinated seed contains between 65%–89% outcrossed progenies in different populations (Mimura *et al.* 2009). In addition, biparental inbreeding affects between 4% and 11% of the progenies (Mimura *et al.* 2009). All *Eucalyptus* species, including *E. globulus*, are believed to have the same chromosome number (2n = 22) (Oudjehih and Bentouati 2006).

The study was based on a Gunns Ltd family trial of *E. globulus* planted in 1989 at Latrobe in northern Tasmania (41° 16’ S, 146° 27’ E). The 570 families used in the trial were from single-tree, open-pollinated seed lots collected from a range-wide base population sampling of *E. globulus*. These families have been assigned to a geographic hierarchy of races, subraces and localities by Dutkowski and Potts (1999). This study focused on variation at the subrace and family levels, consistent with previous studies of this trial (Stackpole *et al.* 2010a,b). The trial design was a resolvable incomplete block design (Patterson and Williams 1976). The trial had five replicates, each divided into 24 incomplete blocks, each of which contained 24 families planted in two-tree plots. The trees that were alive in the trial at age 16 years were measured for diameter at breast height over bark at 1.3 m above ground level (DBH). Of these, a sample of 2163 trees was selected for wood property sampling, omitting four minor subraces (Wilson’s Promontory Lighthouse, Mount Dromedary, Recherche Bay and Western Tasmania) (see Dutkowski and Potts 1999). Four or five individual trees were sampled from each of the 467 families that had four or more suitable candidate trees (alive and more than 10.0 cm in DBH) in the trial. In 452 families, one tree per replicate was sampled while in 12 families only two to three replicates were sampled. The number of families per subrace averaged 27, and ranged from 3 to 107 (see supporting information). Further sampling details are given in Stackpole *et al.* (2010a,b).

### Measurement of wood properties

Cambium to cambium wood cores were removed at 1.1 m above ground level. Each core was cut in half longitudinally and one half used to measure wood density (see Stackpole *et al.* 2010a) and the other half air-dried for predicting wood chemical composition (see Stackpole *et al.* 2010b). We used NIR spectroscopy to estimate wood chemical components as this is the only practical method for measuring the large number of samples required for detailed quantitative genetic analyses. NIR is widely used in assaying wood chemical composition in trees (Tsuchikawa 2007), including eucalypts (Schimleck *et al.* 2000; Raymond and Schimleck 2002). The air-dried wood was ground to pass through a 1-mm screen, and NIR spectra collected using a Bruker Optics Co. MPA (see supporting information). NIR models detailed in the supporting information were used to obtain predictions of S/G (coefficient of determination, *R^2^* = 58.3), Klason lignin (*R^2^* = 66.3), and extractives (*R^2^* = 78.2%) for the 2163 trees. Validation of predictions was undertaken using chemical assays from 45 samples independent from those used to develop the model (S/G *R^2^* = 47.0%, Klason lignin *R^2^* = 60.0%, and extractives *R^2^* = 83.3%). Because of the low phenotypic *R^2^* for S/G, the genetic correlations were calculated between the NIR predictions and the 180 samples measured directly using pyrolysis, and a very high correlation was found (*r*_a_ = 0.99). This means that with the averaging which occurs across families, there is a marked increase in the reliability of the NIR predictions at the genetic level over that at the individual phenotypic level (see supporting information for a more detailed explanation). Cellulose content (validation *R^2^* = 85.0%) and pulp yield (validation *R^2^* = 82.0%) for the same trees was obtained in a similar manner (see Stackpole *et al.* 2010b). Depending upon trait, the number of individuals for which wood property data were available ranged from 2140 to 2163 due to missing values (Table 1).

**Table 1:** Genetic parameters for wood chemical traits in *Eucalyptus globulus*

Trait	n	Mean	*F_subrace_*	*V_a_* (SE)*^a^*	*CV_s_*	*CV_a_*	*h^2^_op_* (SE)	*Q*_ST_ (SE)	*P (Q*_ST_ *>F*_ST_)	Regression of Trait on Latitude		
										β	Significance	*R^2^* (%)
S/G	2149	1.97	15.8^***^	0.002 (0.0003)	4.05	2.55	0.44 (0.056)	0.34 (0.091)	***	0.040	***	76
Klason lignin	2158	20.5	25.2^***^	0.094 (0.019)	2.54	1.50	0.27 (0.052)	0.37 (0.101)	***	−0.117	NS	16
Cellulose*^b^*	2154	43.4	32.4^***^	0.325 (0.048)	2.12	1.31	0.42 (0.056)	0.34 (0.092)	***	0.290	*	30
Extractives	2140	4.76	28.9^***^	0.113 (0.026)	13.7	7.08	0.25 (0.052)	0.44 (0.108)	***	−0.199	*	28
Diameter*^b^*	3383	17.3	2.4^**^	1.535 (0.318)	3.35	7.16	0.15 (0.031)	0.04 (0.026)	NS	0.057	NS	2
Density*^b^*	2145	539	16.4^***^	220.2 (28.2)	3.03	2.75	0.51 (0.058)	0.20 (0.066)	**	−6.560	**	49
Pulp yield*^b^*	2163	53.2	35.5^***^	0.399 (0.061)	2.05	1.19	0.39 (0.055)	0.37 (0.096)	***	0.400	**	41

Included are the number of samples (n); grand mean for each trait; *F_subrace_* value and significance for the difference between subraces; additive genetic variation component (*V_a_*) and its standard error (SE); coefficients of variation at the subrace (*CV_s_*) and additive genetic (*CV_a_*) level; narrow-sense heritability (*h^2^_op_*); quantitative divergence between subraces (*Q*_ST_) and probability (*P*) that *Q*_ST_ > *F*_ST_ of Steane *et al.* (2006); and the slope (β), significance, and coefficient of determination (*R^2^*) for the regression of subrace means on subrace latitude.

^a^ All variance components (V_a_) are significant at *P* < 0.001.

^b^ These traits are from Stackpole *et al.* (2010a)

### Statistical analyses

Following the approach described in Stackpole *et al.* (2010b), a mixed model was fitted to the data from all the races used in the study. Replicate was fitted as a fixed effect, incomplete block and family within-subrace terms fitted as random effects, and subrace fitted as a fixed effect except in the bivariate analyses used to estimate subrace and family correlations. Univariate and bivariate models were fitted with ASReml (Gilmour *et al.* 2001). The effect of the wood chemical traits on survival were tested through calculation of the genetic correlation between the wood chemical and the whole trial survival data (0 = dead, 1 = alive) at age 16 years following the approach of Chambers *et al.* (1996). In addition to the analyses and tests described in Stackpole *et al.* (2010a,b), quantitative genetic divergence between subraces was also assessed using *Q*_ST_; which was calculated following Latta (1998) and Yang *et al.* (1996) as:

$$Q_{ST}=\sigma_{\mathrm{subrace}}^{2}/(\sigma_{\mathrm{subrace}}^{2}+2^{*}\sigma_{\mathrm{add}(\mathrm{subrace})}^{2})$$

where σ^2^_subrace_ is the restricted maximum likelihood estimate of the between subrace variance component, and σ^2^_add(subrace)_ is the estimate of the pooled within-subrace additive genetic variance. σ^2^_add(subrace)_ was calculated from the family within subrace variance component, σ^2^_family(subrace)_ (Stackpole *et al.* 2010b) using a coefficient of relatedness (*r*) of 0.4 for the open-pollinated families. Narrow-sense heritabilities (*h^2^_op_*), the coefficient of additive genetic variance (*CV*_a_), the coefficient of subrace genetic variance (*CV*_s_), subrace genetic correlations, and additive within subrace genetic correlations were calculated as detailed in Stackpole *et al.* (2010b). It should be noted that we expect that the genetic parameters for the chemical traits are likely to be underestimated as the error of NIR prediction is essentially included in the error variance and this would decrease heritability.

The standard errors of *Q*_ST_ and *h^2^_op_* were calculated with ASReml using an expanded Taylor series (Gilmour *et al.* 2001). For each trait a one-tailed likelihood ratio test was used to test whether *Q*_ST_ was greater than the *F*_ST_ derived from putatively neutral microsatellite markers. *Q*_ST_ is the quantitative genetic equivalent to the molecular measure of population divergence *F*_ST_. If *F*_ST_ is measured using selectively neutral markers, then *F*_ST_ is the measure of the genetic differentiation among populations due to random drift or mutation (Latta 1998). If *Q*_ST_ is significantly higher than *F*_ST_, then this is evidence for diversifying natural selection acting on the quantitative trait (Latta 1998; Steane *et al.* 2006). The *F*_ST_ estimates used for this comparison were based on the average racial divergence in eight microsatellite loci as published by Steane *et al.* (2006) (*F*_ST_ = 0.09). We also tested against the highest *F*_ST_ reported for a single microsatellite locus in this species (*F*_ST_ = 0.158; Astorga *et al.* 2004) and obtained identical results but at the 0.05 level of significance.

Correlations were derived from bivariate analyses as the multivariate models with more than two traits did not converge. The difference of the subrace and additive genetic correlations from zero was tested using two-tailed log likelihood tests. The subrace means of each trait were regressed against their latitude of origin. Locality means of extractives and Klason lignin content were also regressed against wood decay reported in Poke *et al.* (2006) and Hamilton *et al.* (2007) which were in common with the present study.

## Results

### Genetic variation within subraces

Highly significant (*P* < 0.001) levels of additive genetic variation were evident within subraces for all traits assessed (LRT of σ^2^_family(subrace)_; Table 1). Diameter had the lowest *h^2^_op_* (0.15 ± 0.03) and density the highest (0.51 ± 0.06), with the wood chemical traits all intermediate. The *h^2^_op_* of the four wood chemical traits (S/G, Klason lignin, extractives and cellulose) ranged from 0.25 to 0.44, and averaged 0.34. S/G had the highest estimate (0.44 ± 0.06) among these wood chemical traits. Despite their relatively high heritabilities, the coefficient of additive genetic variation within subraces (*CV_a_*) for the chemical and physical wood property traits were low when compared with diameter, except for extractives (Table 1). While the *h^2^_op_* of pulp yield was intermediate, it had the lowest *CV_a_* of all traits assessed.

Within subraces, there were strong additive genetic correlations among the wood chemical traits. Genetic variation in Klason lignin was negatively correlated with cellulose (*r*_a_ = −0.90 ± 0.04) (Table 2), weakly negatively correlated with S/G (*r*_a_ = −0.31 ± 0.11), and positively correlated with extractives (*r*_a_ = 0.62 ± 0.10). The S/G was negatively genetically correlated with extractives (*r*_a_ = −0.59 ± 0.10). The additive genetic correlations of chemical traits with growth were statistically significant (*P* < 0.05) but were generally low. Faster growing trees (larger diameter) had less lignin (*r*_a_ = −0.38 ± 0.15), higher S/G (*r*_a_ = 0.33 ± 0.124), and higher cellulose (*r*_a_ = 0.45 ± 0.12) than slower growing trees. No significant correlation between survival and wood chemical traits was detected at either the additive genetic or subrace levels (data not shown). Additive genetic variation in density was weakly negatively correlated with that of Klason lignin (*r*_a_ = −0.23 ± 0.11) and S/G (*r*_a_ = −0.28 ± 0.09). Pulp yield was strongly positively correlated with cellulose (*r*_a_ = 0.91 ± 0.02) and strongly negatively correlated with Klason lignin (*r*_a_ = −0.92 ± 0.04). Higher pulp yield was moderately associated with higher S/G (*r*_a_ = 0.47 ± 0.08), faster growth (diameter, *r*_a_ = 0.53 ± 0.12) and lower extractives (*r*_a_ = −0.61 ± 0.09).

**Table 2:** Correlations among traits in *Eucalyptus globulus* at the additive (*r_a_*), subrace (*r_s_*) and phenotypic (*r_p_*) levels

		S/G	Extractives	Cellulose	Pulp yield	Diameter	Density
Klason lignin	*r_a_*	−0.31^**^	0.62^***^	−0.90^***^	−0.92^***^	−0.38^*^	−0.23^*^
	*r_s_*	−0.56^*^	0.90^***^	−0.98^***^	−0.95^***^	−0.32	0.45
	*r_p_*	−0.41^***^	0.69^***^	−0.86^***^	−0.82^***^	0.10^***^	−0.02
S/G	*r_a_*		−0.59^***^	0.45^***^	0.47^***^	0.33^*^	−0.28^**^
	*r_s_*		−0.78^***^	0.73^**^	0.79^***^	0.02	−0.72^**^
	*r_p_*		−0.49^***^	0.55^***^	0.57^***^	−0.03	−0.30^***^
Extractives	*r_a_*			−0.68^***^	−0.61^***^	−0.29	0.03
	*r_s_*			−0.93^***^	−0.95^***^	−0.07	0.58^*^
	*r_p_*			−0.74^***^	−0.66^***^	0.20^***^	0.25^***^
Cellulose	*r_a_*				0.91^***^	0.45^***^	0.18
	*r_s_*				0.99^***^	0.30	−0.51
	*r_p_*				0.90^***^	0.04	−0.07^**^
Pulp yield	*r_a_*					0.53^***^	0.17
	*r_s_*					0.33	−0.58^*^
	*r_p_*					0.07^**^	−0.07^***^
Diameter	*r_a_*						0.06
	*r_s_*						0.05
	*r_p_*						0.03

The significance of the correlation from zero is indicated (^*^P < 0.05, ^**^P < 0.01, ^***^P < 0.001).

### Subrace level genetic variation

In addition to the significant additive genetic variation within subraces, there were highly significant differences between the subraces of *E. globulus* for all wood chemical traits as well as density, diameter, and pulp yield (Table 1). The level of differentiation between subraces for diameter was significant but low, consistent with its low heritability. The coefficient of subrace variation (*CV_s_*) ranged from 2.05 for pulp yield to an atypical high of 13.7 for extractives. When viewed relative to the additive genetic variation within populations (as measured by either *Q*_ST_ or *CV_s_*/*CV_a_*), the level of genetic variation between subraces for all wood chemical traits was markedly higher than that observed for diameter, and even density (Table 1). *Q*_ST_ was significantly (*P* < 0.001) greater than the race level divergence in neutral molecular markers measured by *F*_ST_ for all the wood chemical traits (Table 1).

At the subrace level, the patterns of variation in the four wood chemical traits (Klason lignin, S/G, extractives and cellulose) were not independent, with all *r*_s_ estimates significant (*P* < 0.05) and most above |0.7| in magnitude (Table 2). The subrace differences in Klason lignin were negatively correlated with S/G (*r*_s_ = −0.56 ± 0.11) and cellulose (*r*_s_ = −0.98 ± 0.03) and positively correlated with extractives (*r*_s_ = 0.90 ± 0.06). Of the wood chemical traits, S/G appeared to be the most genetically independent at the subrace and additive genetic level (Table 2). The subrace variation in S/G was negatively correlated with extractives (*r*_s_ = −0.78 ± 0.11) and positively correlated with cellulose (*r*_s_ = 0.73 ± 0.13).

The subraces showed broad-scale geographic structure in the wood chemical traits (Figure 1); as for cellulose (Stackpole *et al.* 2010b). The main differentiation was between the mainland and Tasmanian subraces. The linear regression of subrace means on subrace latitude of origin was significant for S/G (*R^2^* = 76%, *P* < 0.001), density (*R^2^* = 49%, *P* < 0.01), cellulose (*R^2^* = 30%, *P* < 0.05) and extractives (*R^2^ =* 28%, *P* < 0.05) (Table 1), consistent with latitudinal clines in these traits to varying degrees. The strongest latitudinal cline was with S/G, which tended to increase southward (Figure 1). The highest S/G values (2.10–2.07) occurred in southeastern Tasmania and the inland northeastern Tasmania, southern Tasmania, and Tasman Peninsula subraces and, excluding the last due to small number of families sampled, their subrace means were significantly (*P* < 0.05) higher than all other subraces (see supporting information). The lowest subrace means (range, 1.82–1.96) were from the mainland Victorian subraces from the Otways, Strzelecki Ranges, and Gippsland. Most subraces with intermediate S/G (1.99–2.02; Figure 1) were from the Bass Strait islands (King Island, Furneaux and South Furneaux). Notable deviations from this latitudinal cline were significant differences in S/G between geographically proximal subraces in Victoria (Strzelecki Foothills 1.93 *vs.* Strzelecki Ranges, 1.85; Gippsland Coastal Plain, 1.82) and Tasmania (inland northeastern Tasmania 2.08 *vs.* northeastern Tasmania 1.96).

While extractives had a higher coefficient of variation between subraces (*CV_s_*) than S/G, only a small fraction of this variation could be explained by the latitudinal cline. Extractives content was highest in the Victorian and northeastern Tasmanian subraces (range, 4.9–5.6; except for Gippsland Foothills; seesupporting information) and lowest in subraces from the south of Tasmania (3.7–4.3; Figure 1c). King Island was also notable for its low extractives (3.9). Of particular note is the significantly high extractives content in the two coastal northeastern Tasmanian races (St. Helens, 5.6; inland northeastern Tasmania, 5.5) compared with all other eastern Tasmanian subraces (3.7–4.4) as well as the subraces immediately northward on the Furneaux Islands (Southern Furneaux, 4.2; Flinders Island, 4.4; see supporting information).

Klason lignin showed no significant latitudinal trend over the full geographic range (Table 1; Figure 1), even though there was a weak subrace correlation with S/G (Table 2). As with extractives, Klason lignin increased northward along the continuous distribution of *E. globulus* on the eastern Tasmanian seaboard (Figure 1). While the magnitude of the differences was not large, the Klason lignin of the three northern Tasmanian subraces (St. Helens, 21.3, northeastern Tasmania, 20.8; inland northeastern Tasmania, 20.8) was significantly greater than that of the south-eastern Tasmania (20.2) and southern Tasmania (19.6) subraces (see supporting information).

## Discussion

A key finding of our study is the significant genetic variation in wood chemical composition which occurs between the subraces of *E. globulus*. These subraces clearly differ in Klason lignin content. The pattern of variation observed was similar to that reported by Poke *et al.* (2006) from a different field site in Tasmania (Pearson correlation among the nine localities in common; *r* = 0.7, *P* < 0.05), indicating relatively stable genetic differences between localities across sites. There are few forest tree studies of genetic variation in lignin content and these focus on conifers (Schutt 1958 cited in Zobel and Jett 1995; Sewell *et al.* 2002; Wainhouse *et al.* 1998). In *Picea sitchensis*, bark lignin content displayed a latitudinal trend, thought to be associated with resistance to pests and pathogens (Wainhouse *et al.* 1998). Broad-scale provenance variation in lignin content has also been demonstrated in a large-scale study of the native American grass *Panicum virgatum* (Casler 2005; Casler *et al.* 2004). No significant latitudinal trend was detected for lignin content in the present study, but one was detected for S/G.

There is some evidence that this clinal decrease in S/G with decreasing latitude within *E. globulus* may be part of a much broader continent-wide trend that transgresses multiple eucalypt species. First, while the wood specimens for each species were derived from different sites, Rencoret *et al.* (2008) reported that the S/G of *E. globulus* (at 2.6) was higher than that of species naturally distributed to its north, *viz*: *E. maidenii* (2.0), *E. nitens* (2.1), and particularly *E. grandis* (1.9). Second, del Rio *et al.* (2005) reported that the S/G of *E. globulus* (4.9) was higher than that of the closely related but more northerly distributed *E. pseudoglobulus* (3.7). Third, these trends are also evident across less-related species sampled from native forest from Tasmania to Papua New Guinea (Kawamura and Bland 1967). Despite these studies using different S/G analytical procedures that can give different results, *E. globulus* always had higher S/G than the species distributed to its north in a given study. There is continuous molecular and morphological variation from Tasmanian *E. globulus* to the closely related, northerly *E. bicostata* and *E. pseudoglobulus*, and the low S/G in the mainland subraces of *E. globulus* is potentially reflective of their intermediate status (Jones 2009). As with S/G, there is also the possibility that extractives content between eucalypt species increase northward across the climatic gradient between 40° S and 25° S. In *E. globulus*, for example, in a common environment trial it was shown that the extractives content of the wood from the closely related and more northerly distributed *E. bicostata* (5.7 ± 1.2) and *E. maidenii* (6.7 ± 1.0) was higher than that of *E. globulus* (3.7 ± 1.1) (Miranda and Pereira 2001), which again could reflect an extension of the intraspecific cline observed in extractives.

There are two lines of evidence to indicate that the broad-scale pattern of genetic differentiation in the various wood components of *E. globulus* is a result of divergent natural selection across the geographic range of the species. First, the quantitative differentiation as measured by *Q*_ST_ is significantly greater than that of the neutral marker *F*_ST_ for all wood chemical components, which suggests that subrace divergence has been driven by natural (diversifying) selection (Latta 1998). Second, the broad-scale trends discussed above for S/G and extractives, and the significant associations between latitude of subrace origin and S/G, shows that the genetic differentiation parallels a broad-scale climatic/environmental gradient (Aitken 2004). The observed genetic variation in wood chemical composition may be due to an evolutionary response to abiotic or biotic stresses acting singly or simultaneously (Roelofs *et al.* 2008). For instance, variation in lignin content is likely to be of adaptive importance (Gonzalez-Martinez *et al.* 2006), as it has primary roles in stem strength (Coleman *et al.* 2008), maintenance of water conduction (Gindl 2001; Plomion *et al.* 2001; Voelker 2009; Walker 2006), and possibly defense (Blanchette 1991; Campbell and Sederoff 1996; Del Río *et al.* 2002; Coleman *et al.* 2008; Salmore and Hunter 2001; Schwarze *et al.* 2000; Syafii *et al.* 1988; Wainhouse and Ashburner 1996), each of which are probably affected by spatially varying selection pressures. There is also evidence that variation in S/G may similarly be of adaptive significance (Anterola and Lewis 2002; Coleman *et al.* 2008; Walker 2006). For example, guaiacyl is preferentially deposited in the walls of vessels (Watanabe *et al.* 2004; Wu *et al.* 1992), an arrangement that may exploit its higher hydrophobicity compared with syringyl, and is thought to confer higher hydrostatic impermeability to the vessel wall (Walker 2006). Genetically modified *Populus* genotypes with a higher proportion of guaiacyl have demonstrated increased resistance to breaking of the water column in the vessels (embolism) following water stress (Anterola and Lewis 2002; Coleman *et al.* 2008).

As with lignin, wood extractives are also thought to play a role in the tree defense against pathogens (Boddy 2001; Gierlinger *et al.* 2004; Taylor *et al.* 2002). To test for a geographic relationship between decay and wood chemical composition, published mean wood decay of *E. globulus* at the subrace level, available from Hamilton *et al.* (2007), and the locality means available from Poke *et al.* (2006) were regressed against lignin, extractives, and S/G in the present trial. The regressions were generally not significant; however, there was a single significant negative association (*R^2^* = 65%; *P* < 0.05) between extractives levels from the present study and the locality level wood decay of Poke *et al.* (2006). This is a reasonable correlation for traits across two different trials conducted some years apart. However, as wood decay risk is likely to be higher in wetter climates, it will be challenging to unravel the roles of biotic and abiotic factors in shaping the natural patterns of genetic variation in wood chemical composition (Armbruster and Schwaegerle 1996). In addition, identifying which traits are under selection is complicated by the fact that the chemical traits are genetically correlated with each other, as well as with wood density and growth (see also Poke *et al.* 2006).

The pulpwood breeding objective for *E. globulus* aims to minimize the cost of pulp production per hectare by improving growth rate, density, and pulp yield (Greaves *et al.* 1997). While not currently considered breeding objectives, low total lignin and high S/G (Del Rio *et al.* 2005; Guerra *et al.* 2008; Macleod 2007; Pinto *et al.* 2002) are linked to more efficient chemical pulping, and these traits could be used as selection traits (Clarke 2009). Our study shows that there is significant additive genetic variation in these breeding objective and wood chemical traits, indicating their potential for genetic improvement through both between and within subrace selection. Within subraces, our additive genetic correlations indicate that selecting for increased growth will result in weak correlated genetic responses in wood chemistry, both increasing cellulose and S/G and decreasing lignin content. The positive additive genetic correlation observed between diameter and cellulose (*r_a_* = 0.45) was consistent with that of Apiolaza *et al.* (2005) (*r*_a_ = 0.61 ns) and the average correlation of *r_a_* = 0.56 for five sites of *E. nitens* (Hamilton and Potts 2008), but was substantially different from the negative correlations (*r_a_* -0.16 to -0.43) previously reported by Raymond *et al.* (2001) in *E. globulus*. A previous small-scale study in *E. globulus* did not detect a significant additive genetic correlation between growth and lignin (Poke *et al.* 2006). However, the significant negative genetic correlation in our study (*r_a_* = −0.38) is informative given that two quantitative trait loci (QTL) for lignin have been shown to colocate with QTL for growth in hybrid eucalypts (Kirst *et al.* 2004). A higher rate of lignin production was associated with slower growth, possibly due to competition between the traits for carbon-based products.

Our results suggest that the only correlated response expected from selection for increased wood density within *E. globulus* subraces is a tendency for lignin and S/G to decrease. A negative phenotypic correlation between S/G and wood density was found in *E. globulus* by da Seca and Domingues (2006), a result that occurred at the phenotypic, additive genetic, and subrace levels in the present study. No additive genetic relationship was observed between density and extractives, similar to previous studies that also did not find a genetic (Miranda and Pereira 2002; Poke *et al.* 2006) or phenotypic (Ona *et al.* 1998) correlation. The present study also indicated that selection for increased pulp yield would result in increased cellulose content and S/G but reduced lignin and extractives content and higher S/G. The high subrace and additive genetic correlations between pulp yield and cellulose content demonstrate that they are effectively the same trait (Stackpole *et al.* 2010b). This is consistent with QTL studies in *E. globulus*, where all QTL that were identified for pulp yield colocated with cellulose QTL, although not all cellulose QTL colocated with QTL for pulp yield (Freeman *et al.* 2009; Thamarus *et al.* 2004). A significant negative genetic correlation of pulp yield and cellulose with Klason lignin has been reported previously in *E. globulus* (Poke *et al.* 2006). A negative phenotypic correlation has also been reported between Klason lignin and cellulose content (Ona *et al.* 1998). Such a negative relationship is expected due to the physical complementarity of cellulose and lignin in wood structure (Plomion *et al.* 2001).

In conclusion, our large-scale study has shown significant genetic variation in wood chemical composition at two-geographic scales within the native gene pool of *E. globulus*. There is evidence that this variation may be an adaptive response to either biotic or abiotic factors, although unraveling the nature of this selection will be challenging due to the strong correlation among traits and potential for correlation among the environmental selection agents across the geographic range of the species. Regardless of the cause of the patterns of genetic variation, the genetic correlations observed are generally favorable for a pulp wood breeding objective. This applies both among the chemical traits themselves as well as their correlation with the main breeding objective traits of growth, wood density and pulp yield. However, a future challenge will be to determine whether breeding objectives for adaptation to specific environments (*e.g.*, drier or high disease risk areas) will be compatible with industrial objectives for the improvement of wood properties.

## Supplementary Material

Supporting Information

## References

[bib1] Aitken S. N., 2004 Genecology and adaptation of forest trees, pp. 197–204 in *Encyclopedia of Forest Sciences*, edited by Evans J. Burley J. Youngquist J. Elsevier, Amsterdam

[bib2] Alves A. Schwanninger M. Pereira H. Rodrigues J., 2006 Calibration of NIR to assess lignin composition (H/G ratio) in maritime pine wood using analytical pyrolysis as the reference method. Holzforschung 60: 29–31

[bib3] Anterola A. M. Lewis N. G., 2002 Trends in lignin modification: A comprehensive analysis of the effects of genetic manipulations/mutations on lignification and vascular integrity. Phytochemistry 61: 221–294 12359514 10.1016/s0031-9422(02)00211-x

[bib4] Apiolaza L. A. Raymond C. A. Yeo B. J., 2005 Genetic variation of physical and chemical wood properties of *Eucalyptus globulus*. Silvae Genet. 54: 160–166

[bib5] Armbruster W., and K. Schwaegerle, 1996 Causes of covariation of phenotypic traits among populations. J. Evol. Biol. 9: 261–276

[bib6] Astorga R Soria F. Basurco F. Toval G., 2004 Diversity analysis and genetic structure of *Eucalyptus globulus* Labill, pp. 351–363 in Eucalyptus in a Changing World, edited by Borralho N. M. G. Pereira J. S. Marques C. Coutinho J. Madeira M. RAIZ, Instituto Investigação de Floresta e Papel, Aveiro, Portugal

[bib7] Blanchette R., 1991 Delignification by wood-decay fungi. Annu. Rev. Phytopathol. 29: 381–398

[bib8] Boddy L., 2001 Fungal community ecology and wood decomposition processes in angiosperms: from standing tree to complete decay of coarse woody debris. Ecol. Bull. 49: 43–56

[bib9] Bose S. K. Francis R. C. Govender M. Bush T. Spark A., 2009 Lignin content *vs.* syringyl to guaiacyl ratio amongst poplars. Bioresour. Technol. 100: 1628–1633 18954979 10.1016/j.biortech.2008.08.046

[bib10] Bozell J., 2010 Connecting biomass and petroleum processing with a chemical bridge. Science 329: 522–523 20671177 10.1126/science.1191662

[bib11] Brooker M., 2000 A new classification of the genus *Eucalyptus* L'Her. (Myrtaceae). Aust. Syst. Bot. 13: 79–148

[bib12] Campbell M. Sederoff R., 1996 Variation in lignin content and composition: mechanisms for control and implications for the genetic improvement of plants. Plant Physiol. 110: 3–13 12226169 10.1104/pp.110.1.3 PMC157688

[bib13] Casler M., 2005 Ecotypic variation among switchgrass populations in northern USA. Crop Sci. 45: 388–398

[bib14] Casler M. Vogel K. Taliaferro C. Wynia R., 2004 Latitudinal adaptation of switchgrass populations. Crop Sci. 44: 293–303

[bib15] Chambers P. G. S. Borralho N. M. G. Potts B. M., 1996 Genetic analysis of survival in *Eucalyptus globulus* ssp. *globulus*. Silvae Genet. 45: 107–112

[bib16] Clarke C. R. E., 2009 The profitable pulp mill, in *Australian Forest Genetics Conference*. Forest Products Commission, Fremantle, WA, Australia

[bib17] Coleman H. D. Samuels A. L. Guy R. D. Mansfield S. D., 2008 Perturbed lignification impacts tree growth in hybrid poplar - a function of sink strength, vascular integrity, and photosynthetic assimilation. Plant Physiol. 148: 1229–1237 18805953 10.1104/pp.108.125500 PMC2577275

[bib18] Cotterill, P., and A. Brolin, 1997 Improving *Eucalyptus* wood, pulp and paper quality by genetic selection. Conferencia IUFRO sobre Silvicultura e Melhoramento de Eucaliptos, Salvador, Brazil, August 24–29, 1997, pp. 1–13.

[bib19] da Seca A. M. L. Domingues F. M. J., 2006 Basic density and pulp yield relationship with some chemical parameters in *Eucalyptus* trees. Pesquisa Agropecu. Bras. 41: 1687–1691

[bib20] da Silva Perez D. Guillemain A. Alazard P. Plomion C. Rozenberg P., 2007 Improvement of *Pinus pinaster* Ait. elite trees selection by combining near infrared spectroscopy and genetic tools. Holzforschung 61: 611–622

[bib21] del Río J. Speranza M. Gutiérrez A. Martínez M. Martínez A., 2002 Lignin attack during eucalypt wood decay by selected basidiomycetes: a Py-GC/MS study. J. Anal. Appl. Pyrolysis 64: 421–431

[bib22] del Rio J. C. Gutierrez A. Hernando M. Landin P. Romero J., 2005 Determining the influence of eucalypt lignin composition in paper pulp yield using Py-GC/MS. J. Anal. Appl. Pyrolysis 74: 110–115

[bib92] Dutkowski G. W., 1995 Genetic variation in drought susceptibility of Eucalyptus globulus ssp globulus in plantations in Western Australia, pp. 199–203 in Eucalypt Plantations: Improving Fibre Yield and Quality, edited by Potts B. M. Borralho N. M. G. Reid J. B. Cromer R. N. Tibbits W. N. CRC for Temperate Hardwood Forestry, Hobart, Australia

[bib23] Dutkowski G. W. Potts B. M., 1999 Geographic patterns of genetic variation in *Eucalyptus globulus* ssp *globulus* and a revised racial classification. Aust. J. Bot. 47: 237–263

[bib24] Falconer D. S. Mackay T. F. C., 1996 *Introduction to Quantitative Genetics*. Longman, Harlow, UK

[bib93] FAO 2007 State of the World’s Forests. Food and Agriculture Organization, Rome, 144 pp.

[bib25] Freeman J. S. Whittock S. P. Potts B. M. Vaillancourt R. E., 2009 QTL influencing growth and wood properties in *Eucalyptus globulus*. Tree Genet. Genomes 5: 713–722

[bib26] Gierlinger N. Jacques D. Schwanninger M. Wimmer R. Paques L., 2004 Heartwood extractives and lignin content of different larch species (*Larix* spp.) and relationships to brown-rot decay resistance. Trees (Berl.) 18: 230–236

[bib27] Gilmour A. R. Thompson R. Cullis B. R. Welham S. J., 2001 *ASREML Reference Manual*. NSW Agriculture, Orange, NSW

[bib28] Gindl W., 2001 Cell-wall lignin content related to tracheid dimensions in drought sensitive Austrian pine (*Pinus nigra*). IAWA J. 22: 113–120

[bib29] Godoy E. Rodrigues J. Alves A. Lazo D., 2007 Content and quality study of the lignin by analytical pyrolysis in *Pinus caribaea*. Maderas-Ciencia Y Tecnologia 9: 179–188

[bib30] Gonzalez-Martinez S. Krutovsky K. Neale D., 2006 Forest tree population genomics and adaptive evolution. New Phytol. 170: 227–238 16608450 10.1111/j.1469-8137.2006.01686.x

[bib31] Greaves B. L. Borralho N. M. G. Raymond C. A., 1997 Breeding objective for plantation eucalypts grown for production of kraft pulp. For. Sci. 43: 465–472

[bib32] Guerra A. Elissetche J. Norambuena M. Freer J. Valenzuela S., 2008 Influence of lignin structural features on *Eucalyptus globulus* kraft pulping. Ind. Eng. Chem. Res. 47: 8542–8549

[bib33] Gutierrez A. del Rio J. C. Martinez M. J. Martinez A. T., 1999 Fungal degradation of lipophilic extractives in *Eucalyptus globulus* wood. Appl. Environ. Microbiol. 65: 1367–1371 10103223 10.1128/aem.65.4.1367-1371.1999 PMC91193

[bib34] Hamilton M. G. Greaves B. L. Potts B. M. Dutkowski G. W., 2007 Patterns of longitudinal within-tree variation in pulpwood and solidwood traits differ among *Eucalyptus globulus* genotypes. Ann. For. Sci. 64: 831–837

[bib35] Hamilton M. G. Potts B. M., 2008 *Eucalyptus nitens* genetic parameters. N. Z. J. For. Sci. 38: 102–119

[bib36] Hatfield R. Fukushima R., 2005 Can lignin be accurately measured? Crop Sci. 45: 832–839

[bib37] Hillis W. Sumimoto M., 1989 Effect of extractives on pulping. In: *Natural Products of Woody Plants*, pp. 880–920, edited by Rowe J. Springer, Berlin, Heidelberg, New York

[bib39] Jones, R., 2009 Molecular evolution and genetic control of flowering in *Eucalyptus globulus* species complex. Ph.D. Thesis, School of Plant Sciences, University of Tasmania, Hobart, Tasmania, Australia

[bib40] Kawamura I. Bland D., 1967 The lignins of *Eucalyptus* wood from tropical and temperate zones. Holzforschung 21: 65–74

[bib41] Kirst M. Myburg A. A. De Leon J. P. G. Kirst M. E. Scott J., 2004 Coordinated genetic regulation of growth and lignin revealed by quantitative trait locus analysis of cDNA microarray data in an interspecific backcross of *Eucalyptus*. Plant Physiol. 135: 2368–2378 15299141 10.1104/pp.103.037960 PMC520804

[bib42] Latta R., 1998 Differentiation of allelic frequencies at quantitative trait loci affecting locally adaptive traits. Am. Nat. 151: 283–292 18811359 10.1086/286119

[bib43] Lewis N. G. Yamamoto E., 1990 Lignin: Occurrence, biogenesis and biodegradation. Annu. Rev. Plant Physiol. Plant Mol. Biol. 41: 455–496 11543592 10.1146/annurev.pp.41.060190.002323

[bib44] MacLeod, M., 2007 The top ten factors in kraft pulp yield. Paperi ja Puu (Paper and Timber) 89: 1–7

[bib45] Mimura M. Barbour R. C. Potts B. M. Vaillancourt R. E. Watanabe K. N., 2009 Comparison of contemporary mating patterns in continuous and fragmented *Eucalyptus globulus* native forests. Mol. Ecol. 18: 4180–4192 19769693 10.1111/j.1365-294X.2009.04350.x

[bib46] Miranda I. Pereira H., 2001 Provenance effect on wood chemical composition and pulp yield for *Eucalyptus globulus* Labill. Appita J. 54: 347–351

[bib47] Miranda I. Pereira H., 2002 Variation of pulpwood quality with provenances and site in *Eucalyptus globulus*. Ann. For. Sci. 59: 283–291

[bib48] Ona T. Sonoda T. Ito K. Shibata M., 1998 Relations between various extracted basic densities and wood chemical components in *Eucalyptus globulus*. J. Wood Sci. 44: 165–168

[bib49] Oudjehih B. Bentouati A., 2006 Chromosome numbers of the 59 species of *Eucalyptus* L'Herit. (Myrtaceae). Caryologia 59: 207–212

[bib50] Patterson H.D. Williams E. R, 1976 A new class of resolvable incomplete block designs. Biometrika 63: 83–92

[bib51] Paues N., 1999 *Celbi*, *Leirosa Figueira da Foz—a Swedish Pioneer of Eucalyptus Paper Pulp*. StoraEnso, Stockholm, Sweden

[bib52] Pinto P. C. Evtuguin D. V. Pascoal C. N. Silvestre A. J. D., 2002 Behavior of *Eucalyptus globulus* lignin during kraft pulping. I. Analysis by chemical degradation methods. J. Wood Chem. Technol. 22: 93–108

[bib53] Plomion C. Leprovost G. Stokes A., 2001 Wood formation in trees. Plant Physiol. 127: 1513–1523 11743096 PMC1540185

[bib54] Poke F. S. Potts B. M. Vaillancourt R. E. Raymond C. A., 2006 Genetic parameters for lignin, extractives and decay in *Eucalyptus globulus*. Ann. For. Sci. 63: 1–9

[bib55] Potts B. M. Vaillancourt R. E. Jordan G. Dutkowski G. W. Costa e Silva J., 2004 Exploration of the *Eucalyptus globulus* gene pool, pp. 46–61 in Eucalyptus in a Changing World, edited by Borralho N. M. G. Pereira J. S. Marques C. M. P. Coutinho J. Madeira M., IUFRO, Aviero, Portugal

[bib56] Raymond C. A. Schimleck L. R., 2002 Development of near infrared reflectance analysis calibrations for estimating genetic parameters for cellulose content in *Eucalyptus globulus*. Can. J. For. Res. 32: 170–176

[bib57] Raymond C. A. Schimleck L. R. Muneri A. Michell A. J., 2001 Genetic parameters and genotype-by-environment interactions for pulp yield predicted using near infrared reflectance analysis and pulp productivity in *Eucalyptus globulus*. For. Genet. 8: 213–224

[bib58] Rencoret J. Gutierrez A. del Rio J., 2007 Lipid and lignin composition of woods from different eucalypt species. Holzforschung 61: 165–174

[bib59] Rencoret J. Marques G. Gutiérrez A. Ibarra D. Li J., 2008 Structural characterization of milled wood lignins from different eucalypt species. Holzforschung 62: 514–526

[bib60] Rodrigues J. Graca J. Pereira H., 2001 Influence of tree eccentric growth on syringyl/guaiacyl ratio in *Eucalyptus globulus* wood lignin assessed by analytical pyrolysis. J. Anal. Appl. Pyrolysis 58: 481–489

[bib61] Rodrigues J. Meier D. Faix O. Pereira H., 1999 Determination of tree to tree variation in syringyl/guaiacyl ratio of *Eucalyptus globulus* wood lignin by analytical pyrolysis. J. Anal. Appl. Pyrolysis 48: 121–128

[bib62] Roelofs D. Aarts M. G. M. Schat H. van Straalen N. M., 2008 Functional ecological genomics to demonstrate general and specific responses to abiotic stress. Funct. Ecol. 22: 8–18

[bib63] Salmore A. K. Hunter M. D., 2001 Elevational trends in defense chemistry, vegetation, and reproduction in *Sanguinaria canadensis*. J. Chem. Ecol. 27: 1713–1727 11545366 10.1023/a:1010411122739

[bib64] Schimleck L. R. Raymond C. A. Beadle C. L. Downes G. M. Kube P. D., 2000 Applications of NIR spectroscopy to forest research. Appita Journal 53: 458–464

[bib65] Schwarze F. Baum S. Fink S., 2000 Resistance of fibre regions in wood of *Acer pseudoplatanus* degraded by *Armillaria mellea*. Mycol. Res. 104: 1126–1132

[bib66] Sedjo R., 1993 The carbon cycle and global forest ecosystem. Water Air Soil Pollut. 70: 295–307

[bib67] Sewell M. Davis M. Tuskan G. Wheeler N. Elam C., 2002 Identification of QTLs influencing wood property traits in loblolly pine (*Pinus taeda* L.) II. Chemical wood properties. Theor. Appl. Genet. 104: 214–222 12582689 10.1007/s001220100697

[bib68] Stackpole D. J. Vaillancourt R. E. de Aguigar M. Potts B. M., 2010 a Age trends in genetic parameters for growth and wood density in *Eucalyptus globulus*. Tree Genet. Genomes 6: 179–193

[bib69] Stackpole D. J. Vaillancourt R. E. Downes G. Harwood C. E. Potts B. M., 2010 b Genetic control of kraft pulp yield in *Eucalyptus globulus*. Can. J. For. Res. 50: 917–927

[bib70] Steane D. A. Conod N. Jones R. C. Vaillancourt R. E. Potts B. M., 2006 A comparative analysis of population structure of a forest tree, *Eucalyptus globulus* (Myrtaceae), using microsatellite markers and quantitative traits. Tree Genet. Genomes 2: 30–38

[bib71] Syafii W. Yoshimoto T. Samejima M., 1988 The effect of lignin structure on decay resistance of some tropical woods. Bulletin Tokyo University Forestry 80: 69–77

[bib72] Taylor A. M. Gartner B. L. Morrell J. J., 2002 Heartwood formation and natural durability - A review. Wood and Fiber Science 34: 587–611

[bib73] Thamarus K. Groom K. Bradley A. Raymond C. A. Schimleck L. R., 2004 Identification of quantitative trait loci for wood and fibre properties in two full-sib pedigrees of *Eucalyptus globulus*. Theor. Appl. Genet. 109: 856–864 15133606 10.1007/s00122-004-1699-4

[bib74] Tibbits W. White T. Hodge G. Borralho N., 2006 Genetic variability in freezing tolerance of *Eucalyptus globulus* ssp *globulus* assessed by artificial freezing in winter. Aust. J. Bot. 54: 521–529

[bib75] Toro, M. A., L. Silió, M. C. Rodriguez, F. Soria, and G. Toval, 1998 Genetic analysis of survival to drought in *Eucalyptus globulus* in Spain. Proceedings of the 6th World Congress on Genetics Applied to Livestock Production, Armidale, NSW, Australia, Vol. 27, pp. 499–502

[bib76] Tsuchikawa S. 2007 A review of recent near infrared research for wood and paper. Appl. Spectrosc. Rev. 42: 43–71

[bib77] Turner S. Somerville C., 1997 Collapsed xylem phenotype of Arabidopsis identifies mutants deficient in cellulose deposition in the secondary cell wall. Plant Cell 9: 689–701 9165747 10.1105/tpc.9.5.689 PMC156949

[bib78] Voelker, S. L., 2009 Functional decreases in hydraulic and mechanical properties of field-grown transgenic poplar trees caused by modification of the lignin synthesis pathway through downregulation of the 4-coumarate:coenzyme A ligase gene. Ph.D. Thesis, Oregon State University, Corvallis, Oregon

[bib79] Wainhouse D. Ashburner R., 1996 The influence of genetic and environmental factors on a quantitative defensive trait in spruce. Funct. Ecol. 10: 137–143

[bib80] Wainhouse D. Ashburner R. Ward E. Boswell R., 1998 The effect of lignin and bark wounding on susceptibility of spruce trees to *Dendroctonus micans*. J. Chem. Ecol. 24: 1551–1561

[bib81] Walker J. C. F., 2006 *Primary Wood Processing: Principles and Practice*. Springer, Netherlands

[bib82] Wallis A. F. A. Wearne R. H. Wright P. J., 1996 Analytical characteristics of plantation eucalypt woods relating to kraft pulp yields. Appita 49: 427–432

[bib83] Watanabe Y. Kojima Y. Ona T. Asada T. Sano Y., 2004 Histochemical study on heterogeneity of lignin in *Eucalyptus* species II. The distribution of lignins and polyphenols in the walls of various cell types. IAWA J. 25: 283–295

[bib84] Weng J. Chapple C., 2010 Tansley review: The origin and evolution of lignin biosynthesis. New Phytol. 187: 273–285 20642725 10.1111/j.1469-8137.2010.03327.x

[bib85] Williams K. J. Potts B. M., 1996 The natural distribution of *Eucalyptus* species in Tasmania. Tasforests 8: 39–165

[bib86] Workman J. J., 1992 NIR spectroscopy calibration basics, pp. 247–280 in *Handbook of Near-Infrared Analysis*, edited by Burns D. A. Ciurczak E. W. Marcel Dekker, New York

[bib87] Wu J. Fukuzawa K. Ohtani J., 1992 Distribution of syringyl and guaiacyl lignins in hardwood in relation to habitat and porosity form in wood. Holzforschung 46: 181–185

[bib88] Yang R. Yeh F. Yanchuk A., 1996 A comparison of isozyme and quantitative genetic variation in *Pinus contorta* ssp *latifolia* by FST. Genetics 142: 1045–1052 8849910 10.1093/genetics/142.3.1045 PMC1207004

[bib90] Zobel B. Jett J. B., 1995 *Genetics of Wood Production*. Springer-Verlag, Heidelberg

[bib91] Zobel B. van Buijtenen J., 1989 *Wood Variation: Its Causes and Control*. Springer-Verlag, Berlin, Heidelberg

