# Supplementary material for: Genetic Variation in the Chemical Components of Eucalyptus globulus Wood
Source: G3 (Bethesda). 2011 Jul 1;1(2):151–9. doi: 10.1534/g3.111.000372 (PMC3276126; doi:10.1534/g3.111.000372)
Supplement: Supporting Information [file supp_1_2_151__index.html]

Supporting Information 

# Genetic Variation in the Chemical Components of *Eucalyptus globulus* Wood

## Supporting Information for Stackpole *et al.*, 2011

**Files in this Data Supplement:**

- File S1 - Supporting Material (PDF, 96 KB)
