# Supplementary material for: Genetic Variation in the Chemical Components of Eucalyptus globulus Wood
Source: G3 (Bethesda). 2011 Jul 1;1(2):151–9. doi: 10.1534/g3.111.000372 (PMC3276126; doi:10.1534/g3.111.000372)
Supplement: Supporting Information [file supp_1.2.151_FileS1.pdf]

File S1

Supporting Material

Assessment of wood chemical content using NIR spectroscopy

Wood chemical composition (methanol extractives, Klason lignin, S/G) was assessed using NIR spectroscopy. The advantages and limitations of NIR spectroscopy to assess wood properties are well established by comprehensive reviews (SO *et al.* 2004; TSUCHIKAWA 2007; WORKMAN 2001). The 2170 wood cores sampled from the field trial were air-dried, ground to pass through a 1 mm screen, and their NIR spectra collected without the removal of extractives (POKE *et al.* 2004) using a Bruker Optics Co. MPA. Spectra were recorded with an integration sphere from 12,500 to 3,600 cm^{-1} (800-2778 nm) with a 8 cm^{-1} spectral resolution and 32 scans per spectrum. These NIR spectra were used to predict Klason lignin and extractives content reported as a percentage of original wood sample with models developed by Poke *et al.* (POKE *et al.* 2004). Validation of these predictions was undertaken using chemical assays from 15-21 samples independent from those used to develop the model (Table S1). Cellulose and pulp yield were predicted using NIR model and validation as detailed in STACKPOLE *et al.* (2010).

NIR models were developed to predict S/G using chemical data derived from 180 samples representing all of the subraces sampled. S/G was assayed by analytical pyrolysis combined with high resolution capillary gas chromatography (Py-GC/FID) as detailed by RODRIGUES *et al.* (1999). With this process the levels of S and G were quantified from their degradation products obtained by flame ionisation detection of the extractives-free ground wood. The content of S and G in each sample was calculated as the sum of their identified degradation products in the pyrogram. The NIR models were developed using 135 randomly selected samples; the remaining 45 samples were used for validation (see Table S1). The NIR models were developed with the OPUS version 5.5 software package (Bruker Optik, Ettlingen Germany). The optimal model was identified using cross validation by varying the pre-processing of the spectra, wavelength segment and the rank (number of principal components) of the model. The selection of the optimal model was based upon having a low standard error of cross validation (SECV), a high R^2 and a medium range rank. Validation statistics for the chosen model was obtained by fitting the independent data set to the selected model, yielding the standard error of prediction (SEP) and the associated R^2 (WORKMAN 1992). The R^2 values for cross validation and independent validation of the S/G model were relatively low (Table S1) but the additive genetic (r_a) and subrace (r_s) correlations between the pyrolysis data and the NIR predictions were effectively one (r_a = 0.99). This means that with the averaging which occurs across families and subraces, there is a marked increase in the reliability of the NIR predictions at the genetic level over that at the individual phenotypic level.

Table S1 Cross (CV) and test set (TSV) validations of NIR models used to predict S/G, Klason lignin, and extractives for *Eucalyptus globulus*.

Compound	S/G	Klason lignin	Methanol extractives
Model code	CV354	KLIG_CV93	EXTR220307
Cross validation statistics			
No. samples used for building model	135	45	40
Spectra pre-processing applied to optimal spectra	1st derivative and MSC	Straight line subtraction	Vector normalisation
Spectral regions used in the model (wavenumbers cm^{-1})	6102-5774 4601-4246	12492-4246	12493-4246
No. of principal components used	5	7	7
SECV (root mean square error of cross validation)	0.121	0.701	0.551
R^2 (squared coefficient of determination indicating model fit)	58.3	66.3	78.2
Test set validation statistics			
No. samples used for validation	45	15	21
No. of principal components in the TSV	5	7	7
SEP (Standard error of prediction)	0.124	0.800	0.526
R^2	47.0	60.0	83.3

Subrace least-square means and data

The trial and germplasm studied is being used for commercial tree breeding purposes. Data reported is subject to pre-exiting data exchange agreements and currently can only be made publically available in summary form (Table S2; see also STACKPOLE *et al.* 2010). The corresponding author should be contacted if there is interest in use of the raw data for collaborative research purposes.

Table S2 Subrace means of S/G, Klason lignin, and extractives for *Eucalyptus globulus*

Subrace	Subrace code	No. of families	S/G		Klason lignin		Extractives	
Tasman Peninsula	TP	3	2.10	-	19.9	-	4.1	-
South-eastern Tasmania	SET	53	2.08	a	20.2	d	4.3	c
Inland North-eastern Tasmania	INET	18	2.08	a	20.8	bc	4.4	c
Southern Tasmania	ST	25	2.07	a	19.6	f	3.7	e
Southern Furneaux	SF	45	2.02	b	20.1	e	4.2	cd
St Helens	StH	9	2.00	bc	21.3	a	5.6	a
Flinders Island	FI	48	1.99	bc	20.2	d	4.4	c
King Island	KI	29	1.99	bc	19.4	f	3.9	d
Gippsland Foothills	GF	3	1.97	-	20.2	-	4.2	-
North-eastern Tasmania	NET	16	1.96	cd	20.8	b	5.5	a
Strzelecki Foothills	StF	6	1.93	de	20.7	bc	5.2	ab
Western Otways	WOt	107	1.93	de	20.6	c	5.1	b
Cape Patton	CP	16	1.91	e	20.7	bc	5.2	ab
Eastern Otways	EOt	23	1.90	e	20.9	ab	5.5	a
Far West Otways	FWOt	3	1.88	-	20.7	-	5.9	-
Strzelecki Ranges	StrR	57	1.85	f	21.2	a	5.5	a
Gippsland Coastal Plain	GCP	6	1.82	f	20.7	bc	4.9	b

Subraces are ranked by decreasing S/G. Values within a trait with different appended letters are different at $P < 0.05$ level according to Tukey's tests. '-' represents subraces that were excluded from the multiple comparisons due to small number (3) of families. The geographic position of each subrace is shown in Figure 1 of paper.

References

POKE, F. S., J. K. WRIGHT and C. A. RAYMOND, 2004 Predicting extractives and lignin contents in *Eucalyptus globulus* using near infrared reflectance analysis. Journal of Wood Chemistry and Technology **24:** 55-67.

RODRIGUES, J., D. MEIER, O. FAIX and H. PEREIRA, 1999 Determination of tree to tree variation in syringyl/guaiacyl ratio of *Eucalyptus globulus* wood lignin by analytical pyrolysis. Journal of Analytical and Applied Pyrolysis **48:** 121-128.

SO C. L., B. K. VIA , L. H. GROOM, L. R. SCHIMLECK , T. F. SHUPE, S. S. KELLEY, T. G. RIALS, 2004 Near infrared spectroscopy in the forest products industry. Forest Products Journal **54:** 6-16.

STACKPOLE, D. J., R. E. VAILLANCOURT, G. DOWNES, C. E. HARWOOD and B. M. POTTS, 2010 Genetic control of kraft pulp yield in *Eucalyptus globulus*. Canadian Journal of Forest Research **50:** 917-927.

TSUCHIKAWA S., 2007 A review of recent near infrared research for wood and paper. Applied Spectroscopy Reviews 42: 43-71.

WORKMAN J. J., 2001 Infrared and Raman spectroscopy in paper and pulp analysis. Applied Spectroscopy Reviews 36: 139-168

WORKMAN, J. J., 1992 NIR spectroscopy calibration basics, pp. 247-280 in *Handbook of near-infrared analysis*, edited by D. A. BURNS and E. W. CIURCZAK. M. Dekker, New York.
